# Comparison of Tooth Size Measurements in Orthodontics Using Conventional and 3D Digital Study Models

**DOI:** 10.3390/jcm13030730

**Published:** 2024-01-26

**Authors:** Valentina Petrović, Martina Šlaj, Mia Buljan, Tadej Čivljak, Ana Zulijani, Berislav Perić

**Affiliations:** 1 Private Dental Practice, 10000 Zagreb, Croatia; dr.valentinapetrovic@gmail.com; 2 School of Dental Medicine, University of Zagreb, 10000 Zagreb, Croatia; mslaj@sfzg.hr (M.Š.); pericb@kbd.hr (B.P.); 3 General Hospital ‘Dr Josip Benčević’, 35000 Slavonski Brod, Croatia; buljanmia@gmail.com; 4 Dental Polyclinic Zagreb, 10000 Zagreb, Croatia; civljaktadej@gmail.com; 5 Department of Oral Surgery, Clinical Hospital Center Rijeka, 51000 Rijeka, Croatia; 6 Department of Dental Medicine, Faculty of Dental Medicine and Health, Josip Juraj Strossmayer University of Osijek, 31000 Osijek, Croatia; 7 Department of Oral and Maxillofacial Surgery, University Hospital Dubrava, 10000 Zagreb, Croatia

**Keywords:** orthodontic variables, orthodontic models, 3D digital models, plaster models, accuracy, reproducibility, reliability

## Abstract

(1) **Background:** The objective of this study was to assess which digitization method produces the biggest deviation in the 3D images of tooth size from plaster models made using alginate impressions, which are considered the gold standard in orthodontics. (2) **Methods:** The sample used in this study included 30 subjects (10 males and 20 females). Measurements were made on four types of models: (1) digital models obtained through intraoral scanning and digitized models of plaster cast made from (2) alginate impressions, (3) silicone impressions, and (4) conventional plaster models. Mesio-distal (MD) and buccal/labial–lingual/palatal (BL) dimensions were measured on the reference teeth of the right side of the jaw (central incisor, canine, first premolar, and first molar). Comparisons of tooth size between the methods were conducted using a repeated measurement analysis of variance and the Friedman test, while the intraclass correlation coefficient was used to determine agreement between the different methods. (3) **Results:** The results showed a similar level of agreement between the conventional and digital models in both jaws and the anterior, middle, and posterior segments. Better agreement was found for the MD measurements (r = 0.337–0.798; *p* ≤ 0.05) compared to the BL measurements (r = 0.016–0.542), with a smaller mean difference for MD (0.001–0.50 mm) compared to BL (0.02–1.48 mm) and a smaller measurement error for MD (0.20–0.39) compared to BL (0.38–0.89). There was more frequently a better level of agreement between 3D images than measurements made using a digital caliper on the plaster models with 3D images. (4) **Conclusions:** The differences in measurements between the digital models and conventional plaster models were small and clinically acceptable.

## 1. Introduction

Orthodontics, a specialized field of dentistry, relies on the accurate assessment of dental models for diagnosis and treatment planning. Recently, digital technology has introduced three-dimensional (3D) digital orthodontic models generated through intraoral scanning and computer-aided design and manufacturing (CAD/CAM) techniques. These digital models offer enhanced visualization, precise measurements, efficient storage and retrieval, and the ability to perform virtual simulations and analyses compared to conventional plaster models [1,2,3,4,5].

Orthodontic variables, such as arch widths, tooth dimensions, and craniofacial measurements, are essential for orthodontic assessment. Comparing these variables between conventional and digital models is crucial to assessing the agreement and feasibility of digital models in routine practice. The mesio-distal tooth width, for example, provides important information about spacing, crowding, and Bolton discrepancies.

Studies comparing conventional and digital models have reported mixed results, influenced by scanning technology, software algorithms, image resolution, and operator experience [6,7,8,9].

A systematic review on intraoral scanners concluded that while the accuracy of digital models compared to conventional methods is debatable, they offer advantages in terms of reproducibility, scanning time, patient comfort, and operator experience [9,10].

Previous reviews have shown that digital models demonstrate clinically acceptable precision and accuracy [3,11,12]. They offer benefits in cost, time, space requirements, and diagnostic capabilities, including treatment planning and bracket placement [3,13]. However, landmark identification remains a limitation [3]. Additional studies have highlighted the accuracy and reliability of digital models compared to direct measurements on plaster models, while also noting variability in study quality and inadequate documentation of comparisons between techniques [11].

Intrarater reliability, analyzed in a study by Luu et al., has shown clinically insignificant differences between digital models and plaster models. The review primarily focused on linear measurements, excluding qualitative ordinal measures. These findings suggest that laser-acquired models and CBCT-acquired models can be used as reliable and valid alternatives to traditional plaster models for orthodontic measurements [14].

The objective of this study was to determine which digitalization method results in the biggest deviations in three-dimensional images of orthodontic variables when compared to plaster models obtained from alginate impressions. Specifically, we analyzed the digitized plaster model after alginate and silicone impressions were taken and examined digital images obtained through direct intraoral scanning. By determining which method yields the largest deviation compared to the plaster model obtained after the alginate impression, we aimed to assess the accuracy of these digitization techniques.

## 2. Materials and Methods

This study was conducted according to the guidelines of the Declaration of Helsinki and approved by the Ethics Committee of the School of Dental Medicine, University of Zagreb (05-PA-15-12/2017 on 14 December 2017).

The study included a total of 30 subjects (10 males and 20 females). The minimum sample size was estimated to be 24 subjects, based on an expected difference of 0.5 mm between manual and digital measurements, with a standard deviation of 0.6 mm in each group, a power of 80%, and a significance level of 0.05. All subjects fulfilling the following criteria were included in the study: complete upper and lower dentition, including permanent incisors, canines, premolars, and first molars; teeth with normal morphology; and teeth without clinically visible signs of abrasion, caries, or poorly fitted fillings that could affect the tooth dimensions. A total of 120 models were produced, divided into four groups of 30: plaster models and digitized models of plaster cast from alginate impressions, digitized models of plaster cast made from silicone impressions, and digital models obtained through intraoral scanning.

Impressions of the dental arches of each subject were taken using alginate (Orthotrace alginate, Cavex, Haarlem, The Netherlands) and silicone (3M ESPE, Seefeld, Germany) impression materials, following the guidelines provided by the manufacturers. The alginate impressions were transported to the laboratory in a 100% humidity environment and poured within 30 min to minimize dimensional changes. The models were cast using white orthodontic plaster Sherakfo-gips (Shera, Lemfoerde, Germany), following the manufacturer’s instructions. One hour after this process, the model was removed from the impression and stored at room temperature for 48 h. The plaster models were scanned using a laboratory scanner, namely, the NeWay scanner (Open technologies, Brescia, Italy), in accordance with the manufacturer’s instructions. The intraoral scans of the dental arches were performed using a Trios 3 intraoral scan device (3Shape, Copenhagen, Denmark). 

**Figure 1:**
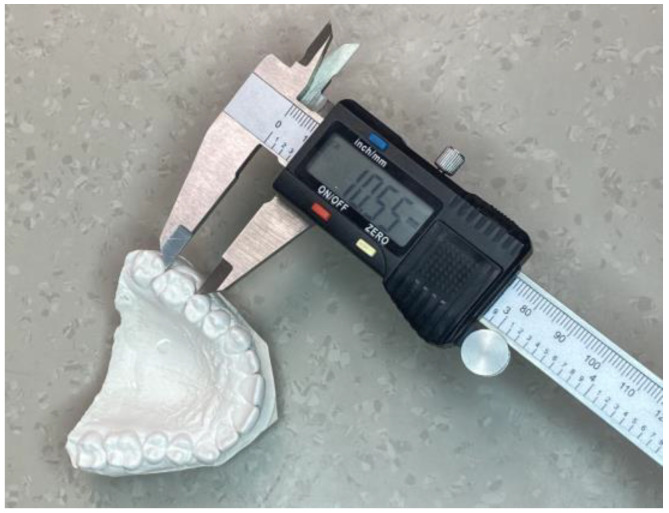
Manual measurement of the plaster models obtained from an alginate impression using a Mitutoyo digital caliper.

**Figure 2:**
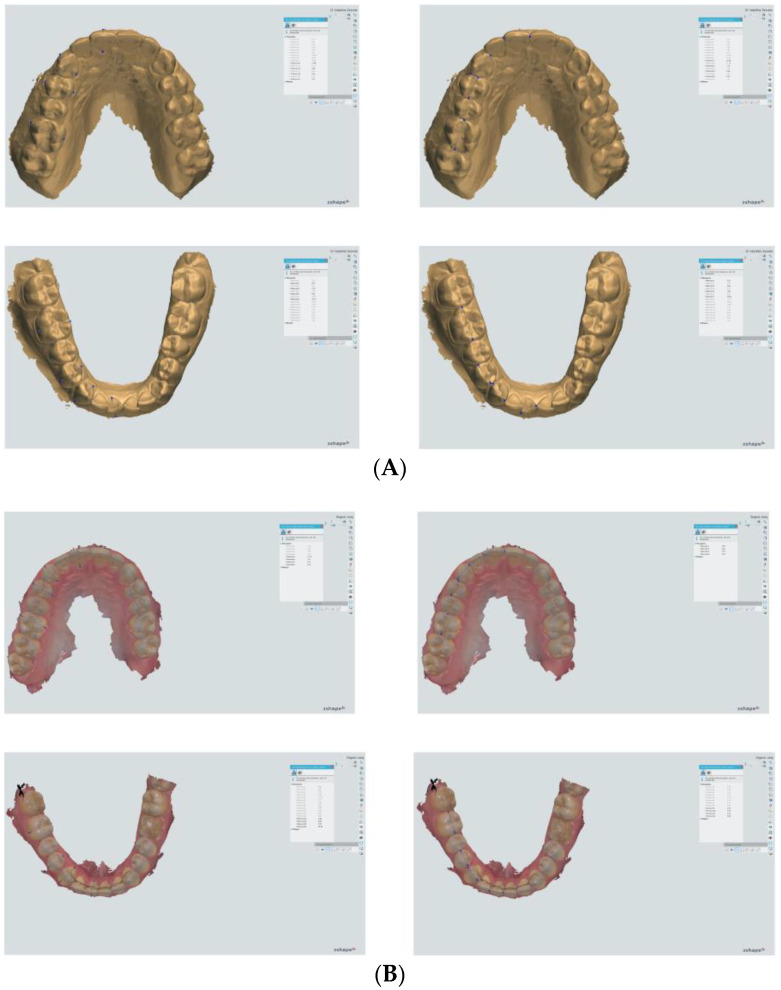
The measurements of mesio-distal and buccal/labial–lingual/palatal dimensions on both the maxilla and mandible (central incisor, canine, first premolar, and first molar) using Ortho Analyzer software: (**A**) digitized plaster model and (**B**) intraoral scan.

The measurements of the digital models were performed using Ortho Analyzer software (version 1.5.1.7, 3Shape, Copenhagen, Denmark) and through manual measurements on the plaster models obtained from an alginate impression using a Mitutoyo digital caliper (Mississauga, ON, Canada) (Figure 1 and Figure 2). 

The mesial–distal (MD) and buccal/labial–lingual/palatal (BL) dimensions of the reference teeth (central incisor, canine, first premolar, and first molar) on the right side of the maxilla and mandible were measured (Figure 2). The MD dimension of each tooth was measured at the greatest distance between the mesial and distal contact points. The BL dimension was defined as the widest distance between the buccal/labial and lingual/palatal surfaces. The measurements were made perpendicular to the tooth axis of the individual teeth.

All measurements were conducted simultaneously and independently by two examiners. After one month, the measurements of five randomly selected models were repeated by both examiners to determine the measurement error using the Dahlberg formula.

After determining the normality of the distribution using the Shapiro–Wilk test, along with descriptive parameters, parametric and non-parametric statistical tests were performed. The intraclass correlation coefficient (ICC) with a 95% confidence interval was used. According to the ICC interpretation criteria, a value less than 0.5 indicates poor reproducibility, 0.5–0.75 is considered moderate, 0.75–0.9 is considered good, and a value greater than 0.9 is considered excellent. The measurement error (ME) was calculated as the square root of the residual variance from the analysis of variance. The smallest detectable change (SDC) was calculated using the following formula: SDC = 1.96 × √2 ME. The concordance of the two measurement methods was determined by calculating the mean value of the measurement differences and constructing a concordance interval (CI), where 95% of the difference between the measurements is located. For this purpose, Blunt–Altman diagrams were made, with the difference in measurements on the *X*-axis and the average of measurements on the *Y*-axis. The limits of agreement (LOAs) were calculated as the average of the pairwise difference ±1.96 * the standard deviation of the difference between the two measurements. A *t*-test was also conducted to analyze the deviation of the mean of the sample from the hypothetical mean, which was set to 0. This determined the significance of any fixed deviation in the measurements. The proportion of cases where the difference between the two types of measurements fell within the matching limits was also quantified. In cases where the *t*-test showed no significant differences, linear regression was performed to analyze the connection between the differences in measurements and the average of the measurements of the two methods. This was performed to check for proportionality bias, i.e., whether one method gives values that are systematically higher (or lower) than those provided by another method.

To compare all four methods simultaneously, the analysis of variance for repeated measurements or the Friedman test was used, and the post hoc Sidak and Wilcoxon tests with Bonferroni correction were also used, depending on the distribution of the data.

Cohen’s criteria were used for interpretation (for r, V, and η): 0.1–0.3 = small effect size, 0.3–0.5 = moderate/large effect size, 0.5–0.7 = large effect size, and >0.7 = very large effect size. For eta-squared: <0.09 = small effect size, 0.09–0.25 = moderate effect size, 0.25–0.49 = large effect size, and >0.49 = very large effect size.

## 3. Results

### 3.1. Reproducibility of Measurements

The differences in the measurements were small and, in most cases, not statistically significant (Table 1). Moreover, the differences in the measurements between the two examiners were small and, in most cases, not statistically significant (*p* > 0.05), and the ones that were statistically significant ranged from 0.2 to 0.6 mm (*p* ≤ 0.028), with a random error of up to 0.021 (Table 2). Smaller differences were observed in the measurements taken from plaster models compared to those taken from intraoral scans (Table 1).

### 3.2. Comparison of Methods for Measuring Molar Dimensions

#### 3.2.1. MD Width of Tooth 16

The measurements on the plaster and digital models showed statistically significant differences (*p* < 0.001; η2 = 0.214), with a range from 0.08 to 0.33 mm. However, there were no significant differences observed between the manual measurements and intraoral scans, although both differed from the digitized models (Table 3). The observed differences between the three methods were not clinically significant, as indicated by a good level of agreement (ICC = 0.800; 95% CI 0.668–0.805; *p* < 0.001) and a small measurement error (ME = 0.27 mm).

When the accuracy of the measurements made on the plaster models was compared to that of the measurements made on each group of digital models, a moderate level of agreement was found (ICC = 0.674–0.725; Table 4). The margin of measurement error, ranging from 0.31 to 0.35 mm, was smaller than the detectable change (0.87–0.96 mm). Notably, a statistically significant fixed deviation of 0.24–0.33 mm was found in the manual measurements when compared to the digitized models of plaster cast made from alginate and silicone impressions, with a hypothetical average deviation of 0 mm (*p* ≤ 0.006). However, the deviation between the manual measurements and intraoral scans was not significant, with it measuring only 0.16 mm.

#### 3.2.2. BL Width of Tooth 16

There was a significant difference (*p* < 0.001; η2 = 0.429) between the manual measurements and the digital measurements obtained from the intraoral scans and digitized models. Compared to the digital measurements, the manual measurements ranged from 0.50 to 0.68 mm (Table 3). However, no significant difference was found among the measurements obtained from the digital models (difference range of 0.07–0.19 mm). The level of agreement between the three methods was poor (ICC = 0.425; 95% CI 0.191–0.642; *p* < 0.001), with a small measurement error (ME = 0.36 mm).

The manual measurements on the plaster models showed poor agreement compared to the digital measurements obtained from each digital model (ICC = 0.149–0.251; Table 4). The measurement error ranged from 0.42 to 0.47 mm, which was smaller than the detectable change (1.17–1.31 mm). A statistically significant fixed bias of 0.50–0.68 mm was found between the manual and digital measurements compared with a hypothetical mean difference of 0 mm (*p* < 0.001).

#### 3.2.3. MD Width of Tooth 46

The measurements showed significant differences between all the models. The manual measurements ranged from 0.20 to 0.31 mm, while the digital measurements showed differences ranging from 0.03 to 0.12 mm (Table 3). The reproducibility of the measurements was found to be good (r = 0.853; 95% CI 0.722–0.926), with a small measurement error of 0.21 mm. The measurement error was larger between the measurements made on the intraoral scan and plaster models (0.27), with reproducibility r = 0.781 (95% CI 0.722–0.926; Table 4).

#### 3.2.4. BL Width of Tooth 46

The measurements showed a significant difference, with the manual measurements differing significantly from the measurements obtained from the digital models in a range of 1.24 to 1.48 mm (*p* < 0.001; Table 3). In contrast, the measurement differences between the digital models were relatively lower, within a range of 0.10 to 0.24 mm. Furthermore, the reproducibility was poor (r = 0.176; 95% CI 0.027–0.377), with a measurement error of 0.65 mm. Notably, when comparing the plaster models to the digital models, significant discrepancies were observed, with a measurement error of 0.9 mm. (Table 4). The reason for this was determined to be random error, as two measurements were recorded as 11 mm, whilst all the others ranged from 6 to 8 mm.

### 3.3. Comparison of Methods for Measuring Premolar Dimensions

#### 3.3.1. MD Width of Tooth 14

The measurements showed a significant difference (*p* < 0.001; η2 = 0.465; Table 3), with the manual measurements differing significantly from the digital measurements in a range of 0.43 to 0.50 mm. In contrast, the differences observed between the digital measurements were not significant (0.01–0.07 mm). The agreement between the three methods was poor (ICC = 0.461; 95% CI 0.207–0.679; *p* < 0.001), with a small measurement error of 0.26 mm. 

Comparing the accuracy of the manual measurements made on the plaster models with the measurements made on each group of digital models, a poor level of agreement was found (ICC = 0.337–0.448; Table 5). The measurement error ranged from 0.34 to 0.39 mm. A statistically significant fixed deviation of 0.43–0.50 mm for the manual measurements compared to the digital measurements was found, with a hypothetical average deviation of 0 mm (*p* < 0.001).

#### 3.3.2. BL Width of Tooth 14

A significant difference between measurements was found (*p* = 0.023; η2 = 0.136) in a range of 0.07–0.26 mm (Table 3). The manual measurements only statistically significantly differed from the measurements obtained from the digitized models of the plaster cast made from alginate impressions. The agreement between the three methods was moderate (ICC = 0.569; 95% CI 0.393–0.734; *p* < 0.001), with a small measurement error of 0.29 mm.

Comparing the accuracy of the manual measurements made on the plaster models to the measurements made on each group of digital models, a poor level of agreement was found (ICC = 0.190–0.234; Table 5). The measurement error ranged from 0.44 to 0.48 mm. A statistically significant deviation of 0.21–0.26 mm was found for the manual measurements compared to the digital measurements on the digitized models of plaster cast made from alginate and silicone impressions, with a hypothetical average deviation of 0 mm (*p* ≤ 0.035); this was not found for the intraoral scans, however, with this value measuring only 0.14 mm.

#### 3.3.3. MD Width of Tooth 44

The differences among all the measurements were not statistically significant (*p* = 0.143; η2 = 0.066; Table 3), with a range of 0.01 to 0.13 mm. The agreement between the three methods was good (ICC = 0.773; 95% CI 0.650–0.871; *p* < 0.001), with a small measurement error of 0.21 mm.

Comparing the difference in the manual measurements with the digital measurements on each group of digital models, the agreement was found to be moderate (ICC = 0.659–0.744; Table 5). The measurement error was small, ranging from 0.23 to 0.27 mm, and deviations of 0.07–0.13 mm were observed in the manual measurements compared to the digital measurements.

#### 3.3.4. BL Width of Tooth 44

The measurement methods were not significantly different (*p* = 0.134; η2 = 0.073; Table 3), with differences ranging from 0.02 to 0.29 mm. The agreement between the three methods was poor (ICC = 0.156; 95% CI 0.004–0.364; *p* = 0.022), with a large measurement error of 0.56 mm.

Comparing the manual measurements on the plaster models with the measurements on each group of digital models, the agreement was poor (ICC = 0.016–0.032; Table 5). The measurement error was found to be large, ranging from 0.75 to 0.77 mm. However, the deviation of the manual measurements from the digital measurements was not significant, ranging from 0.02 to 0.29 mm.

### 3.4. Comparison of Methods for Measuring Canine Dimensions

#### 3.4.1. MD Width of Tooth 13

The measurement methods were significantly different (*p* < 0.001; η2 = 0.316; Table 3), with the manual measurements differing significantly from the measurements obtained from the digital models by 0.22–0.34 mm, while the digital measurements were not significantly different (difference range: 0.01–0.11 mm). The agreement between the three methods was moderate (ICC = 0.602; 95% CI 0.390–0.770; *p* < 0.001), with a small measurement error of 0.23 mm.

Comparing the manual measurements obtained from the plaster models with the measurements obtained from each group of digital models, the agreement was poor to moderate (ICC = 0.420–0.550; Table 6). The measurement error was found to be small, ranging from 0.28 to 0.29 mm. A statistically significant fixed deviation of 0.22 to 0.34 mm was found between the manual measurements and the measurements on the digital models compared to the hypothesized average deviation of 0 mm (*p* ≤ 0.004).

#### 3.4.2. BL Width of Tooth 13

The methods showed significant differences (*p* < 0.001; η2 = 0.446; Table 3), with the manual measurements differing significantly from the digital measurements in a range of 0.34 to 0.79 mm. Additionally, the measurements between each group of digital models also demonstrated significant differences (0.23–0.45 mm). The agreement among the three methods was poor (ICC = 0.324; 95% CI 0.117–0.547; *p* < 0.001), with a small measurement error of 0.38 mm. 

Moreover, when comparing the manual measurements on the plaster models with the measurement on each group of digital models, the agreement was poor (ICC = 0.151–0.324; Table 6). The observed measurement error was small, ranging from 0.38 to 0.53 mm. A statistically significant fixed deviation of 0.34 to 0.79 mm was found between the manual and digital measurements compared to the hypothesized average deviation of 0 mm (*p* ≤ 0.015).

#### 3.4.3. MD Width of Tooth 43

Statistically significant differences were observed between the measurements (*p* < 0.001; Table 3), particularly when comparing the manual measurements and the measurements from the digital models. The correlation coefficient (r) was found to be 0.623 (95% CI 0.386–0.792), with a measurement error of 0.20 mm. The agreement between the measurements on the plaster models with those on the digital models was moderate (r = 0.526–0.544), with a measurement error ranging from 0.20 mm to 0.26 mm (Table 6).

#### 3.4.4. BL Width of Tooth 43

The measurements showed statistically significant differences (*p* < 0.001; Table 3). However, no significant differences were found between the manual measurements and the measurements obtained from the intraoral scans, with a range of 0.03 to 0.51 mm being found. The mean measurement error was 0.37 mm, and the correlation coefficient was moderate (r = 0.568; 95% CI 0.356–0.745). The measurements on the plaster models showed the weakest correlation with the measurements obtained from the digitized models of plaster cast made from silicon impressions (r = 0.405), with a larger error of 0.49 mm and a larger discrepancy in measurements of 0.51 mm being found (Table 6).

### 3.5. Comparison of Methods for Measuring Incisor Dimensions

#### 3.5.1. MD Width of Tooth 11

The measurements showed statistically significant differences (*p* < 0.001; η2 = 0.285; Table 3); the manual measurements on the plaster models were statistically different from the measurements on the digital models. The differences in the measurements ranged from 0.25 to 0.31 mm. Notably, no significant differences were found between the different groups of digital models (range: 0.27–0.62 mm). The agreement among the three methods was considered good (ICC = 0.776; 95% CI 0.617–0.881; *p* < 0.001), with a small measurement error of 0.23 mm.

Comparing the manual measurements on the plaster models with the measurements on each group of digital models, the agreement was moderate (ICC = 0.647–0.661; Table 7). The measurement error was small, ranging from 0.29 to 0.32 mm. A statistically significant fixed deviation of 0.25–0.31 mm was found between the manual measurements and the digital measurements, compared to the hypothesized average deviation of 0 mm (*p* ≤ 0.006).

#### 3.5.2. BL Width of Tooth 11

There were significant differences (*p* < 0.001; η2 = 0.480; Table 3) between the manual measurements and the measurements on each group of digital models, with differences ranging from 0.36 to 0.80 mm. Additionally, significant differences were observed among the digital measurements themselves, with differences in a range of 0.12 to 0.45 mm. The agreement between the three methods was good (ICC = 0.380; 95% CI 0.145–0.607; *p* < 0.001), with a small measurement error of 0.35 mm.

Comparing the manual measurements on the plaster models with the measurements on each group of digital models, the agreement was poor (ICC = 0.190–0.232; Table 7). The measurement error was small, ranging from 0.44 to 0.48 mm. There was also a statistically significant fixed deviation of 0.35–0.80 mm between the manual measurements and the digital measurements compared to the hypothesized average deviation of 0 mm (*p* ≤ 0.007).

#### 3.5.3. MD Width of Tooth 41

The measurement methods showed significant differences, with a small measurement error of 0.20 and moderate repeatability (r = 0.611; 95% 0.440–0.765; Table 3). The weakest repeatability was observed between the plaster and digitized models of plaster cast made from silicone impressions (r = 0.353), with an error of 0.28 mm and a difference in measurements of 0.15 mm (Table 7).

#### 3.5.4. BL Width of Tooth 41

The measurement methods showed significant differences, with poor repeatability (r = 0.440) and a small measurement error of 0.39. The most significant differences were observed between the measurements obtained from the plaster models and the measurements obtained from the digitized models of plaster cast made from silicon impressions, with a deviation of 0.60 mm, an error of 0.51 mm, and a repeatability of 0.342 (Table 2 and Table 7).

These results provide insights into the repeatability and agreement of different measurement methods for various dental dimensions. The differences between the measurement methods were clinically insignificant.

## 4. Discussion

The comparison of orthodontic variables obtained from conventional and 3D digital orthodontic models has been a topic of interest in the orthodontics field for many years. Several studies have investigated the accuracy and reliability of digital models in capturing anatomical structures and dimensions [5,15,16,17,18,19]. Our study has revealed several important findings that shed light on the accuracy and reliability of digital models in orthodontics. These findings have significant implications for the clinical application of digital models and highlight areas where further research is needed.

Firstly, our findings underscore that measurements on plaster models exhibit superior repeatability compared to digital models obtained from intraoral scans, followed by digitized models of plaster cast made from silicone impressions and alginate impressions. This suggests that the type of scanning technique used can significantly impact the repeatability and consistency of measurements. The superior repeatability of the plaster models could be attributed to the direct physical replication of the oral structures, minimizing potential errors associated with data processing and algorithmic calculations inherent in digital models [20,21]. During the intraoral scanning process, errors can be caused by factors such as surrounding soft tissue, the presence of saliva and bleeding, patients’ movements, the size of the scanner head, limited mouth opening, and the experience of the operator [22]. However, the limitations of plaster models, such as time-consuming processes and susceptibility to damage, storage space and condition problems, and data sharing, should also be considered [23,24,25]. Digitized models share some disadvantages with conventional plaster models, including impression (contraction of the impression material) and pouring (expansion of the plaster) process errors [26]. The greater accuracy of the digitized model of plaster cast obtained from silicone impressions compared to alginate impressions can be explained by the higher dimensional stability of the material, with it showing less sensitivity to time-dependent deformation [16,27]. 

Moreover, our study demonstrated a higher level of agreement among the various forms of digital models compared to the agreement observed between the plaster and digital models. This indicates that digital models, regardless of the scanning technique used, tend to produce more consistent results. This may be attributed to the standardized nature of digital scans and the elimination of potential errors associated with the manual manipulation of plaster models during the measurement process, as well as deformation changes in alginate impressions and the pouring process [3,7,28].

Regarding the location of measurements, our results showed a similar level of agreement between conventional and digital models in both the maxilla and mandible. This suggests that the accuracy of digital models is not significantly affected by the anatomical region being evaluated, providing confidence in their use for comprehensive orthodontic assessments. In terms of tooth dimensions, our study found better agreement for the MD measurements compared to the BL measurements. This indicates that digital models may be reliable in accurately capturing the MD aspects of tooth morphology, while caution should be exercised when assessing BL dimensions due to slightly greater biological variability. To achieve more accurate measurements of the MD and BL dimensions, the measurements were performed perpendicular to the tooth axis of each individual tooth. This approach aimed to prevent measurement errors caused by tooth misalignment [29]. The analysis of dental arch sections demonstrated similar levels of agreement for the anterior, middle, and posterior segments. This implies that digital models have an equal ability to capture the overall shape and alignment of teeth throughout the dental arch, regardless of their specific location.

Lastly, our study found that differences in measurement values between conventional and digital models are primarily due to random errors rather than systematic errors. This suggests that any variations between the two methods are likely a result of inherent measurement variability rather than consistent biases, further supporting the reliability of digital models in accurately reflecting dental structures [4,30].

Overall, the outcomes of this study provide strong evidence to support the reliability of digital orthodontic models, including those derived from intraoral scans and digitized plaster models made from silicone impressions, in offering precise measurements of orthodontic variables. Nonetheless, it is crucial for clinicians and researchers to carefully consider the specific variables under assessment and recognize their potential sources of variability. Heightened awareness of both the capabilities and constraints of digital models is imperative when integrating them into orthodontic clinical practice and research.

The present study has several limitations that should be noted. This study does not fully address potential errors that may result from variations in dentist impression-taking techniques, premature measurements of uncooled plaster models, or differences in pouring technique. Additionally, further research on this topic should also consider the influence of soft tissue features. 

## 5. Conclusions

Measurements obtained from plaster models generally exhibit greater repeatability when compared to intraoral scans, followed by digitized models of plaster cast obtained from silicone impressions, and least commonly, those obtained from alginate impressions. Better levels of agreement were more frequently observed among the digital models compared to the measurements taken using a digital caliper on the plaster models. Similar levels of agreement were found between the measurements on the plaster models and digital models in both the maxilla and mandible, as well as in the anterior, middle, and posterior parts of the dental arch. The differences in the measurements are primarily due to random rather than systematic errors.

## Figures and Tables

**Table 1:** Reproducibility of measurements for different models by the same examiner.

	Intraoral Scan (IOS)			Digitized Plaster Model after Alginate Impression (DPMA)			Digitized Plaster Model after Silicone Impression (DPMS)			Plaster Model (PM)		
Parameter	Mean ± SD	*p* *	Dahlberg	Mean ± SD	*p* *	Dahlberg	Mean ± SD	*p* *	Dahlberg	Mean ± SD	*p* *	Dahlberg
MD 16	0.02 ± 0.05	0.434	0.01	−0.12 ± 0.19	0.251	0.04	−0.03 ± 0.06	0.264	0.01	0.06 ± 0.05	0.07	0.02
MD 14	−0.01 ± 0.05	0.81	0.00	−0.04 ± 0.07	0.27	0.01	0.00 ± 0.05	0.874	0.00	0.06 ± 0.05	0.07	0.02
MD 13	0.01 ± 0.05	0.603	0.00	−0.03 ± 0.06	0.276	0.01	−0.04 ± 0.05	0.152	0.01	0.02 ± 0.08	0.621	0.01
MD 11	0.01 ± 0.03	0.485	0.00	−0.06 ± 0.01	<0.001	0.02	−0.03 ± 0.05	0.212	0.01	0.02 ± 0.08	0.621	0.01
BL16	−0.05 ± 0.04	0.079	0.01	−0.04 ± 0.04	0.089	0.01	−0.04 ± 0.03	0.072	0.01	−0.02 ± 0.04	0.374	0.01
BL 14	−0.03 ± 0.05	0.251	0.01	0.00 ± 0.07	0.954	0.00	−0.01 ± 0.06	0.835	0.00	−0.02 ± 0.08	0.621	0.01
BL 13	−0.03 ± 0.04	0.194	0.01	−0.06 ± 0.06	0.115	0.02	−0.03 ± 0.04	0.164	0.01	−0.04 ± 0.05	0.178	0.01
BL 11	−0.05 ± 0.08	0.257	0.02	−0.02 ± 0.06	0.567	0.01	−0.01 ± 0.03	0.363	0.00	0.00 ± 0.07	1	0.00

SD—standard deviation. *p*—significance level. * *t*-test for repeated measurement.

**Table 2:** Reproducibility of measurements for different models between the two examiners.

	Intraoral Scan (IOS)			Digitized Plaster Model after Alginate Impression (DPMA)			Digitized Plaster Model after Silicone Impression (DPMS)			Plaster Model (PM)		
Parameter	Mean ± SD	*p* *	Dahlberg	Mean ± SD	*p* *	Dahlberg	Mean ± SD	*p* *	Dahlberg	Mean ± SD	*p* *	Dahlberg
MD 16	0.21 ± 0.20	0.085	0.07	0.36 ± 0.24	**0.028**	0.11	−0.03 ± 0.09	0.517	0.01	0.02 ± 0.04	0.374	0.01
MD 14	0.31 ± 0.18	**0.017**	0.10	0.12 ± 0.28	0.385	0.04	0.02 ± 0.08	0.655	0.01	0.00 ± 0.00	1	0.00
MD 13	0.32 ± 0.28	0.063	0.10	−0.02 ± 0.22	0.879	0.01	−0.02 ± 0.07	0.533	0.01	0.00 ± 0.07	1	0.00
MD 11	0.02 ± 0.10	0.728	0.01	−0.03 ± 0.20	0.784	0.01	0.01 ± 0.07	0.773	0.00	−0.12 ± 0.18	0.208	0.04
BL16	−0.36 ± 0.20	**0.017**	0.11	−0.12 ± 0.20	0.245	0.04	−0.01 ± 0.06	0.826	0.00	0.00 ± 0.00	1	0.00
BL 14	−0.06 ± 0.26	0.64	0.02	0.07 ± 0.33	0.661	0.02	0.02 ± 0.05	0.394	0.01	0.00 ± 0.00	1	0.00
BL 13	0.40 ± 0.20	**0.011**	0.13	0.21 ± 0.13	**0.022**	0.07	0.01 ± 0.04	0.711	0.00	−0.00 ± 0.00	1	0.00
BL 11	0.31 ± 0.14	**0.008**	0.10	0.44 ± 0.21	**0.009**	0.14	0.04 ± 0.05	0.178	0.01	−0.02 ± 0.04	0.374	0.01

SD—standard deviation. *p*—significance level. * *t*-test for repeated measurement.

**Table 3:** Descriptive analysis of the comparison between the methods used for the reference teeth.

Parameter	IOS AS * ± SD	DPMA AS * ± SD	DPMS AS * ± SD	PM AS * ± SD	*p* **	η2 ***
16 MD	10.40 ^a^ ± 0.64	10.47 ^ab^ ± 0.62	10.56 ^b^ ± 0.70	10.24 ^bc^ ± 0.67	0.001	0.214
16 BL	6.72 ^a^ ± 0.49	6.53 ^a^ ± 0.49	6.61 ^a^ ± 0.56	7.21 ^b^ ± 0.61	<0.001	0.429
46 MD	10.89 ^a^ ± 0.62	10.98 ^ab^ ± 0.56	11.00 ^b^ ± 0.65	10.69 ^c^ ± 0.72	<0.001	0.320
46 BL	5.66 ^a^ ± 0.49	5.41 ^b^ ± 0.50	5.52 ^a^ ± 0.42	6.89 ^c^ ± 1.34	<0.001	0.537
14 MD	7.22 ^a^ ± 0.40	7.28 ^a^ ± 0.35	7.29 ^a^ ± 0.41	6.79 ^b^ ± 0.49	<0.001	0.465
14 BL	5.87 ^a^ ± 0.40	5.75 ^b^ ± 0.37	5.80 ^ab^ ± 0.42	6.01 ^a^ ± 0.58	0.023	0.136
44 MD	7.01 ± 0.41	7.00 ± 0.40	7.01 ± 0.42	6.93 ± 0.53	0.143	0.066
44 BL	4.67 ± 0.39	4.42 ± 0.43	4.40 ± 0.40	4.69 ± 1.03	0.134	0.073
13 MD	8.11 ^a^ ± 0.41	8.21 ^a^ ± 0.39	8.22 ^a^ ± 0.38	7.89 ^b^ ± 0.47	<0.001	0.316
13 BL	7.40 ^a^ ± 0.41	7.62 ^b^ ± 0.47	7.84 ^c^ ± 0.43	7.06 ^a^ ± 0.70	<0.001	0.446
43 MD	6.94 ^a^ ± 0.37	7.05 ^a^ ± 0.33	7.07 ^a^ ± 0.34	6.74 ^b^ ± 0.43	<0.001	0.390
43 BL	6.71 ^a^ ± 0.50	6.98 ^b^ ± 0.55	7.19 ^c^ ± 0.49	6.68 ^ab^ ± 0.86	<0.001	0.310
11 MD	8.71 ^a^ ± 0.53	8.77 ^a^ ± 0.51	8.74 ^a^ ± 0.52	8.46 ^b^ ± 0.64	0.001	0.285
11 BL	6.61 ^a^ ± 0.44	6.73 ^a^ ± 0.49	7.06 ^b^ ± 0.45	6.25 ^c^ ± 0.65	<0.001	0.480
41 MD	5.41 ^a^ ± 0.28	5.51 ^b^ ± 0.32	5.56 ^b^ ± 0.33	5.41 ^ab^ ± 0.37	0.016	0.135
41 BL	5.51 ^a^ ± 0.44	5.74 ^b^ ± 0.38	6.06 ^c^ ± 0.35	5.46 ^ab^ ± 0.91	<0.001	0.338

* methods with different letters in superscripts were statistically significantly different based on the results of the SidakoBLg post hoc test. ** ANOVA for repeated measures. *** effect size.

**Table 4:** Comparison of measurements between the plaster and digital models for tooth 16 and tooth 46.

Parameter	ICC (95% CI)	ME *	Mean Difference (95% CI)	*p*
16 MD				
PM-IOS	0.705 (0.468–0.848)	0.35	−0.16 (−0.34–0.03)	0.087
PM-DPMA	0.725 (0.439–0.868)	0.31	−0.24 (−0.40–(−0.07))	0.006
PM-DPMS	0.674 (0.294–0.850)	0.35	−0.33 (−0.51–(−0.15))	0.001
16 BL				
PM-IOS	0.149 (−0.092–0.481) ^ns^	0.47	0.50 (0.24–0.75)	<0.001
PM-DPMA	0.243 (−0.095–0.554)	0.42	0.68 (0.46–0.91)	<0.001
PM-DPMS	0.251 (−0.078–0.549)	0.46	0.61 (0.36–0.85)	<0.001
46 MD				
PM-IOS	0.781 (0.591–0.912)	0.27	−0.20 (−0.34–(−0.05))	0.008
PM-DPMA	0.797 (0.309–0.925)	0.23	−0.29 (−0.41–(−0.17))	<0.001
PM-DPMS	0.798 (0.276–0.927)	0.24	−0.31 (−0.44–(−0.19))	<0.001
46 BL				
PM-IOS	0.139 (−0.101–0.412) ^ns^	0.88	1.24 (0.77–1.70)	<0.001
PM-DPMA	0.106 (−0.096–0.358) ^ns^	0.89	1.48 (1.00–1.95)	<0.001
PM-DPMS	0.100 (−0.101–0.519) ^ns^	0.89	1.37 (0.90–1.84)	<0.001

* ME—measurement error quantified using the square root of the residual variance. ^ns^—not significant.

**Table 5:** Comparison of measurements between the plaster and digital models of tooth 14 and tooth 44.

Parameter	ICC (95% CI)	ME *	Mean Difference (95% CI)	*p*
14 MD				
PM-IOS	0.337 (0.031–0.639)	0.39	−0.43 (−0.61–(−0.26))	<0.001
PM-DPMA	0.448 (0.110–0.693)	0.34	−0.49 (−0.66–(−0.32))	<0.001
PM-DPMS	0.439 (0.115–0.683)	0.37	−0.50 (−0.66–(−0.34))	<0.001
14 BL				
PM-IOS	0.232 (−0.080–0.522)	0.47	0.14 (−0.07–0.34)	0.186
PM-DPMA	0.234 (−0.072–0.522)	0.48	0.26 (0.07–0.44)	0.007
PM-DPMS	0.190 (−0.098–0.463)	0.44	0.21 (0.02–0.40)	0.035
44 MD				
PM-IOS	0.680 (0.433–0.833)	0.27	−0.08 (−0.22–0.06)	0.263
PM-DPMA	0.659 (0.401–0.821	0.27	−0.07 (−0.22–0.07)	0.309
PM-DPMS	0.744 (0.515–0.872)	0.23	−0.13 (−0.25–(−0.01))	0.035
44 BL				
PM-IOS	0.039 (−0.336–0.395) ^ns^	0.75	0.02 (−0.38–0.42)	0.918
PM-DPMA	0.032 (−0.313–0.376) ^ns^	0.77	0.27 (−0.13–0.67)	0.181
PM-DPMS	0.016 (−0.324–0.361) ^ns^	0.76	0.29 (−0.12–0.69)	0.156

* ME—measurement error quantified using the square root of the residual variance. ^ns^—not significant.

**Table 6:** Comparison of measurements between the plaster and digital models of tooth 13 and tooth 43.

Parameter	ICC (95% CI)	ME *	Mean Difference (95% CI)	*p*
13 MD				
PM-IOS	0.550 (0.201–0.766)	0.28	−0.22 (−0.37–(−0.08))	0.004
PM-DPMA	0.426 (0.017–0.698	0.29	−0.33 (−0.48–(−0.17))	<0.001
PM-DPMS	0.42 (0.003–0.698)	0.29	−0.34 (−0.49–(−0.18))	<0.001
13 BL				
PM-IOS	0.324 (0.117–0.547)	0.38	−0.34 (−0.61–(−0.07))	0.015
PM-DPMA	0.192 (−0.120–0.491)	0.51	−0.57 (0.85–(−0.29))	<0.001
PM-DPMS	0.151 (−0.112–0.433)	0.53	−0.79 (−1.06-(−0.52))	<0.001
43 MD				
PM-IOS	0.530 (0.184–0.753)	0.26	−0.20 (−0.34–(−0.07))	0.004
PM-DPMA	0.526 (−0.027–0.795)	0.21	−0.31 (−0.43–(−0.20))	<0.001
PM-DPMS	0.544 (−0.059–0.816)	0.20	−0.33 (−0.44–(−0.23))	<0.001
43 BL				
PM-IOS	0.542 (0.227–0.753)	0.48	−0.03 (−0.29–0.22)	0.786
PM-DPMA	0.528 (0.211–0.744)	0.47	−0.30 (0.55–0.05)	0.019
PM-DPMS	0.405 (0.021–0.677)	0.49	−0.51 (−0.77–0.25)	<0.001

* ME—measurement error quantified using the square root of the residual variance.

**Table 7:** Comparison of measurements between the plaster and digital models of tooth 11 and tooth 41.

Parameter	ICC (95% CI)	ME *	Mean Difference (95% CI)	*p*
11 MD				
PM-IOS	0.647 (0.327–0.824)	0.32	−0.25 (−0.42–(−0.08))	0.006
PM-DPMA	0.661 (0.214–0.851)	0.29	−0.31 (−0.46–(−0.16))	<0.001
PM-DPMS	0.647 (0.271–0.833)	0.31	−0.28 (−0.45–(−0.12))	0.001
11 BL				
PM-IOS	0.232 (−0.080–0.522)	0.47	−0.35 (−0.60–(−0.10))	0.007
PM-DPMA	0.234 (−0.072–0.522)	0.48	−0.48 (−0.73–(−0.22))	0.001
PM-DPMS	0.190 (−0.098–0.463)	0.44	−0.80 (−1.03–(−0.57))	<0.001
41 MD				
PM-IOS	0.625 (0.342–0.803)	0.21	0.00 (−0.11–0.11)	0.985
PM-DPMA	0.640 (0.370–0.810)	0.20	−0.10 (−0.21–0.01)	0.067
PM-DPMS	0.353 (0.023–0.622)	0.28	−0.15 (−0.30–(−0.01))	0.041
41 BL				
PM-IOS	0.493 (0.162–0.723)	0.48	−0.05 (−0.32–0.23)	0.726
PM-DPMA	0.438 (0.114–0.682)	0.51	−0.28 (−0.55–(−0.01))	0.043
PM-DPMS	0.342 (−0.037–0.632)	0.51	−0.60 (−0.87–(−0.33))	<0.001

* ME—measurement error quantified using the square root of the residual variance.

## Data Availability

Data are not publicly available.
